# The Impact of Young and/or Exercised Blood Plasma Transfusions in Individuals With Neurodegenerative Conditions: Protocol for a Scoping Review

**DOI:** 10.2196/65935

**Published:** 2025-08-19

**Authors:** Michael James Kirton, Emily R Cox, Lance Dalleck, Belinda Lange, Claire Drummond, Ranjay Chakraborty, Alline Beleigoli, Joyce S Ramos

**Affiliations:** 1 College of Nursing and Health Sciences, Caring Futures Institute Flinders University Adelaide, SA Australia; 2 School of Biomedical Sciences and Pharmacy University of Newcastle Callaghan, NSW Australia; 3 Active Living and Learning Research Program Hunter Medical Research Institute New Lambton, NSW Australia; 4 Recreation, Exercise, and Sports Science Department Western Colorado University Gunnison, CO United States; 5 College of Science and Engineering, Medical Device Research Institute Flinders University Adelaide, SA Australia; 6 College of Medicine and Public Health, Rural and Remote Health Flinders University Adelaide, SA Australia; 7 College of Medicine and Public Health, Flinders Health and Medical Research Institute Flinders University Adelaide, SA Australia

**Keywords:** neurodegenerative conditions, plasma transfusion, young plasma, exercised plasma, aged, population, concept, context

## Abstract

**Background:**

The prevalence of neurodegenerative conditions is expected to continue to rise, with current treatments largely focused on the management of symptoms and not disease progression. Exercise is a therapy that has significant favorable effects on several of the modifiable risk factors that influence the development/progression of some neurodegenerative conditions. However, there are significant barriers to exercise for this population, warranting consideration of alternative or adjunct therapies. Blood plasma transfusion is an emerging nonpharmaceutical/nonexercise therapy that may have positive benefits on people and animal models with neurodegenerative conditions.

**Objective:**

The aim of this study was to investigate and synthesize the literature on the impacts of young or exercised blood plasma transfusions in people and animal models with neurodegenerative conditions.

**Methods:**

We will include studies that have explored the impacts of young or exercised blood plasma transfusions in adults or animal models with neurodegenerative conditions. PubMed Central, MEDLINE, Cochrane Database of Systematic Reviews, Scopus, ProQuest, SPORTDiscus, PROSPERO, and multiple unpublished sources, including book chapters, conference papers, and theses, will be searched to include literature from January 2004 to May 2024, written in English. Studies will be screened by 2 independent reviewers; a third reviewer will be used to resolve any conflicts. Data will be extracted into a modified charting tool and will be presented in a graphical or tabular format combined with the appropriate narration.

**Results:**

As of June 2024, the literature search has been completed. After searching listed databases, 3646 citations and 13 gray literature studies were identified, of which 1432 duplicates were removed.

**Conclusions:**

To the best of our knowledge, this study will be the first study with the aim of investigating the literature on the impacts of young or exercised blood plasma transfusions in individuals with neurodegenerative conditions.

**International Registered Report Identifier (IRRID):**

DERR1-10.2196/65935

## Introduction

### Background

Neurodegenerative conditions such as Parkinson disease, Alzheimer disease, motor neuron disease, and multiple sclerosis are a form of neurological condition affecting more than 3 billion people worldwide [1]. With a growing and aging population, the prevalence of neurodegenerative conditions is expected to continue to rise [2]. Neurodegenerative conditions refer to a collection of conditions that are primarily characterized by neuron dysfunction and loss [2]. An important feature of many neurodegenerative conditions is the involvement of proteins with altered physicochemical properties, commonly referred to as misfolded proteins [3], resulting in altered function and/or intracellular or extracellular accumulation of the proteins [2].

Treating these conditions is challenging, as relatively little is known about their etiology and pathophysiology [4]. Therefore, therapeutic interventions typically focus on the management of symptoms or reducing the impact of impairments rather than on disease progression [5]. Nevertheless, there is some evidence showing that the development of some neurodegenerative conditions is a direct or indirect result of modifiable risk factors [6]. Some of these risk factors include diabetes, hypertension, obesity, depression, and physical inactivity [6]. Exercise is a modality that has been shown to have significant favorable effects on all of these modifiable risk factors [6]. Exercise is also a well-established method of positively influencing brain cognition, neurogenesis, and plasticity [7]. However, there are significant barriers to exercise in neurodegenerative populations that lead to poor long-term exercise adherence [8,9]. Barriers include low outcome expectation, fear of falling, functional or physiological impairment, and lack of time [8,9]. Therefore, alternative/adjunct therapies are warranted that may overcome the barriers to exercise while still providing similar benefit.

Blood transfusion of plasma from young (young plasma) or athletic (exercised plasma) individuals is emerging as a potential therapy to alleviate symptoms and potentially progression of some neurodegenerative conditions [6,10,11]. Young plasma transfusions have been shown to be safe and well tolerated in both humans and animals, with minimal adverse events or reactions [12-14]. A study in a mice model has demonstrated that young plasma transfusions can reverse some of the various age-related changes in the mouse brain, including, but not limited to, hippocampal neurogenesis and improved cognition [15]. Similar findings have been reported in clinical human studies with young plasma or therapeutic plasma exchange [16]. Specifically in humans, platelet-rich plasma infusions have been shown to reduce neuroinflammatory compounds, including tumor necrosis factor-α, which has a systemic anti-inflammatory effect [12,17].

Conversely, there is limited research on the safety, tolerability, and impacts of exercised plasma transfusions in human and animal models [6]. Specifically in humans, only 1 study protocol [6] has been published to investigate the safety and efficacy of exercised plasma transfusions in patients with Alzheimer disease. That study is currently in progress and is expected to be completed in 2026 (NCT05068830). There is some animal model research on the safety, tolerability, and impacts of exercised blood plasma transfusions in neurodegenerative conditions [11]. One trial found that exercised blood plasma transfusions from aged and exercised donor mice conferred the beneficial effects of exercise on cognition and hippocampal neurogenesis in aged sedentary mice [11]. Therefore, the aim of this scoping review is to investigate, synthesize, and present the current research literature on the impacts of blood plasma transfusions (young or exercised) in people and animal models with neurodegenerative conditions and to identify gaps in the existing research literature and areas for future inquiry.

A preliminary search of PubMed, the Cochrane Database of Systematic Reviews, Google Scholar, and Joanna Briggs Institute (JBI) Evidence Synthesis was conducted, and no current or underway systematic reviews or scoping reviews on the impacts of young and/or exercised blood plasma transfusions in people or animal models with neurodegenerative conditions were identified. Our preliminary search identified a systematic scoping review, which investigated the safety and efficacy of replacement fluids in therapeutic plasma exchange [16]. However, the impacts of young and/or exercised plasma in neurodegenerative conditions has not been explored in that study [16].

### Review Objectives

The objectives of this scoping review are as follows.

1. Synthesize the literature on the impacts of young or exercised blood plasma transfusions in people and animal models with neurodegenerative conditions.
2. Identify the gaps in the existing literature and areas for future research on the impacts of young or exercised blood plasma transfusions in people and animal models with neurodegenerative conditions.

### Review Questions

The research questions of this scoping review are as follows.

1. What are the impacts of blood plasma transfusions (young or exercised) in people or animal models with neurodegenerative conditions?
2. What is known about the safety and tolerability of young or exercised plasma transfusions in people or animal models with neurodegenerative conditions and what physiological parameters are measured to monitor the safety and tolerability of this therapy?
3. What are the protocols implementing young or exercised plasma transfusions in people or animal models with neurodegenerative conditions (intervention period, dosage, donor parameters)?
4. What are the barriers and facilitators to the implementation of plasma transfusions (young or exercised) in people or animal models with neurodegenerative conditions?
5. What is the cost-effectiveness of young or exercised blood plasma transfusion in people with neurodegenerative conditions?

## Methods

The proposed scoping review will be conducted in accordance with the JBI methodology for scoping reviews and the PRISMA-ScR (Preferred Reporting Items for Systematic reviews and Meta-Analyses extension for Scoping Reviews) guidelines (Multimedia Appendix 1) [18,19].

### Inclusion Criteria

#### Participants

Only studies that included adults or animal models with neurodegenerative conditions will be considered. Adults and animal models with any one of the following neurodegenerative conditions will be included: Parkinson disease, Alzheimer disease, motor neuron disease, vascular dementia, Lewy body dementia, multiple sclerosis, prion disease, amyotrophic lateral sclerosis, Huntington disease, and spinocerebellar ataxia. Additionally, aged adults or animal models will be included, as aging is the single greatest contributing risk factor to the development of neurodegenerative conditions [20].

#### Concepts

The key concepts that will be addressed are the impacts of blood plasma transfusions in individuals with neurodegenerative conditions, plasma exchange/transfusion, young plasma, and exercised plasma. The following are the working definitions of the concepts used in the scoping review.

##### Plasma Exchange/Transfusion

Plasma transfusion is a blood plasma replacement therapy. Typically, patients’ blood is removed via an intravenous needle into an exchange machine via tubing, which separates the various components of blood, including removing the plasma. The exchange machine then adds a plasma substitute, which can include young plasma or exercised plasma. Lastly, the new plasma is returned to the patient through the tubing.

##### Young Plasma/Exercised Plasma

Young plasma is blood plasma drawn from young individuals. In human trials, healthy individuals aged 18-30 years are usually considered young donors [12]. In animal models, specifically mice trials, healthy individuals aged 3-4 months are usually considered young donors [15].

Exercised plasma is blood plasma drawn from athletically fit individuals. As this is a new therapy, there is limited research on donor criteria. However, of the available protocols, donor inclusion criteria includes healthy male donors, aged 18-40 years, with a maximal oxygen uptake of ≥55 mL/kg/min, and a BMI of ≤27 kg/m^2^ [6]. In animal models, specifically mice, donor inclusion criteria included healthy, aged (18 months old) mice who were given continuous access to a running wheel for 6 weeks [11].

#### Context

The context for this scoping review will be the globally published and unpublished research on the impacts of blood plasma (young or exercised) transfusions in people or animal models with neurodegenerative conditions.

### Types of Studies

This scoping review will consider both experimental and quasi-experimental study designs, including controlled and noncontrolled studies (randomized controlled trials, controlled clinical trials, nonrandomized controlled trials, and pre-post studies). Although randomized controlled trials are preferred, other study types will be included, as not all research questions can be answered with a randomized controlled trial. Systematic reviews that meet the inclusion criteria will also be considered depending on the research question. In addition, protocol-only papers will be included to allow a deeper understanding of protocol development. The search for unpublished studies will include book chapters, conference papers, and theses. However, unpublished studies will be excluded if a published study is available. Only studies written in English will be considered due to time and resource constraints. Studies related to the topic published between January 2004 and May 2024 will be considered to capture the modern use of blood plasma transfusions. Papers focused on any of the following aspects of blood plasma transfusion will not be considered.

1. Use of other types of plasma or replacement fluids that are not young or exercised plasma or if the use of young or exercised plasma is not specified.
2. Application in nonneurodegenerative conditions or a neurodegenerative condition that is not listed above.

### Search Strategy

The search strategy will aim to locate both published and unpublished studies. A 3-step search strategy will be utilized.  An initial limited search of PubMed and Google Scholar using keywords (Textbox 1) will be undertaken to identify the studies on the topic. The text words contained in the titles and abstracts as well as the index terms of relevant studies will be used to develop a preliminary search strategy (Multimedia Appendix 2) tailored to each information source.

Subsequently, this preliminary search strategy will be used to search for the following sources of research that will be scoped for this review.

1. Electronic databases: PubMed Central, MEDLINE, Cochrane Database of Systematic Reviews, Scopus, ProQuest, and SPORTDiscus.
2. Trial registries: PROSPERO.
3. Unpublished studies: the search for unpublished studies will include book chapters, conference papers, and theses.

The search strategy, including all the identified keywords and index terms, will be adapted for each included database and/or information source. The reference list of all the included sources of evidence will be screened for additional studies.

**Development of the population, concept, context search terms for the preliminary search.**

**Population**

- Neurological patients
- Neurodegenerative
- Neurological models
- Parkinson disease
- Alzheimer disease
- Motor neuron disease
- Prion disease
- Dementia
- Amyotrophic lateral sclerosis
- Multiple sclerosis
- Huntington disease
- Spinocerebellar ataxia

**Concept**

- Plasma transfusion
- Plasma exchange
- Blood plasma transfusion
- Therapeutic plasma exchange
- Blood component transfusion
- Infusion
- Transfusion medicine
- Plasma infusion
- Plasmapheresis
- Exercised plasma
- Exercise trained donors
- Young plasma
- Young fresh frozen plasma
- Blood factor transfer

**Context**

- Therapeutic effects
- Efficacy
- Effects
- Tolerability
- Rejuvenative
- Improve

### Study Selection

Following the search, all identified citations will be collated and uploaded into Endnote version 21.2/2024 (Clarivate Analytics) and duplicates removed. Titles and abstracts of potentially relevant sources will be retrieved, and their citation details will be imported into the Covidence systematic review software, Veritas Health Innovation. Following a pilot test, titles and abstracts will then be screened by 2 independent reviewers (MJK and ERC) for assessment against the inclusion criteria for the review.

The full text of the selected citations will be assessed in detail against the inclusion criteria by 2 or more independent reviewers. The reasons for exclusion at full text will be recorded. Any disagreements that arise between the reviewers at each stage of the selection process will be resolved through discussion or with an additional reviewer (JSR). The results of the search and the study inclusion process will be reported in full in the final scoping review and presented in a PRISMA flow diagram [19,21].

### Data Extraction

Data will be extracted from the included papers by 2 or more independent reviewers by using the standardized data extraction tools available in JBI SUMARI [18]. The data extracted will include specific details about the participants, concept, context, study methods, interventions, and key findings relevant to the review questions. Based on the contributions of Kohli et al [16] and Teferra et al [22] and considering the keywords, a modified preliminary data extraction tool is proposed (Multimedia Appendix 3). The draft data extraction tool will be modified and revised as necessary during the process of extracting data from each included evidence source. Any disagreements between the reviewers (MJK and ERC) will be resolved through discussion or with an additional reviewer (JSR). Where required, authors of papers will be contacted to request missing or additional data.

### Data Presentation/Analysis

According to the population, concept, context framework (Textbox 1) that has been outlined in this scoping review protocol, the extracted data will be presented in a graphical or tabular format combined with appropriate narration. The extracted information will be presented in categories, as described in Multimedia Appendix 3. If any additional information needs to be categorized, it will be categorized at the time of review. The narrative summary will include the objectives, aims, results of review questions, and concepts. A summary of the findings will be used to provide an overall description of the included studies. The scoping review will address the various elements of the research questions established and identify gaps for further research. The chosen method of data analysis and presentation was selected because of the broad scope of the included participants/studies to allow for multiple types of transfusions and neurodegenerative conditions to be included and the relatively new nature of the field. A PRISMA flow diagram (Multimedia Appendix 4) was modified to report the results of the literature search [19].

## Results

As of June 2024, the literature search has been completed. After searching the listed databases, 3646 citations and 13 gray literature studies were identified, of which 1432 duplicates were removed. Subsequent abstract screening (completed by MJK and ERC) and full-text review (MJK, ERC, JSR) were completed and submitted for publication in June 2025. The search and screening results are summarized in the PRISMA flow diagram (Multimedia Appendix 4) [19].

## Discussion

### Overview

Previous research has demonstrated that young and/or exercised blood plasma transfusions may have therapeutic benefits in people [12] and animal [11] models with neurodegenerative conditions. However, studies report conflicting outcomes [11,12,23]. For example, a recent study showed positive benefits on neurogenesis after the administration of exercised plasma [11]; however, another similar study found no significant benefits on neurogenesis [23]. Thus, the impacts of young and/or exercised blood plasma transfusions in individuals with neurodegenerative conditions remain unclear. Further to this, exercised blood plasma has been postulated to potentially be superior or have a greater effect in certain neurodegenerative conditions (Alzheimer disease) when compared to young blood plasma [6]. The primary aim of this scoping review is to therefore investigate, synthesize, and present the current research on the impacts of blood plasma transfusions (young or exercised) in people and animal models with a neurodegenerative condition. This scoping review will also identify gaps in the existing research literature and areas for future inquiry.

Our results will outline the similarities and differences between the included studies on the impacts of young and/or exercised blood plasma transfusions and identify common trends between them. These factors are important to inform future research and any potential future implementation of young and/or exercised blood plasma transfusions in the treatment/management of neurodegenerative conditions.

To the best of our knowledge, this will be the first study to systematically scope and synthesize the literature on the impacts of young and/or exercised blood plasma transfusions in individuals with neurodegenerative conditions. This scoping review will have several limitations, including omitting studies that do not specify the population, type, or age group of the plasma donors used, which may potentially exclude relevant studies. Additionally, only including studies written in English is another limitation by potentially excluding relevant research published in other languages.

### Conclusion

Overall, this scoping review aims to synthesize the current research on young and/or exercised blood plasma transfusion in people/animal models with neurodegenerative conditions. This work may inform future research and aid in the development of increasing robust studies to elucidate the effectiveness of these types of plasma transfusions in people/animal models with neurodegenerative conditions.

## Appendix

Multimedia Appendix 1 PRISMA-ScR checklist.

Multimedia Appendix 2 Preliminary search strategy for scoping review.

Multimedia Appendix 3 Preliminary data extraction tool.

Multimedia Appendix 4 PRISMA flow diagram: search results and study selection and inclusion process.

## Abbreviations

JBI: Joanna Briggs Institute
PRISMA-ScR: Preferred Reporting Items for Systematic Reviews and Meta-Analyses Extension for Scoping Reviews

## Data Availability

All data relevant to this study are included within this paper or uploaded as Multimedia Appendices 1-4.
