# Supplementary material for: The Impact of Young and/or Exercised Blood Plasma Transfusions in Individuals With Neurodegenerative Conditions: Protocol for a Scoping Review
Source: JMIR Res Protoc. 2025 Aug 19;14:e65935. doi: 10.2196/65935 (PMC12405790; doi:10.2196/65935)
Supplement: Multimedia Appendix 2 [file resprot_v14i1e65935_app2.docx]

Ovid MEDLINE(R) and Epub Ahead of Print, In-Process, In-Data-Review & Other Non-Indexed Citations, Daily and Versions <1946 to May 30, 2024>

| 1 (neurological or neurodegenerative).tw. 405199 |
| --- |
| 2 (Parkinson's disease or Alzheimer's disease or Motor neuron disease or Dementia or Multiple sclerosis or Prion disease or Amyotrophic lateral sclerosis or Huntington's disease or Spinocerebellar ataxia).tw. 488508 |
| 3 ("blood transfusion" or "blood component transfusion" or "plasma transfusion" or plasmapheresis or "plasma exchange" or "plasma infusion" or "transfusion medicine").tw. 64043 |
| 4 ("exercised plasma" or "young plasma" or "young fresh frozen plasma" or "exercise-trained donors" or "exercise trained donors" or "exercise plasma" or "young blood" or "exercise plasma").tw. 890 |
| 5 (restor* or reverse* or rejuvenat* or therapeutic or therap* or "therapeutic effect*" or safe* or efficacy or improve* or progress* or effect* or tolerab* or symptom* or attenuate* or boost* or gut microbio* or "gut-brain axis" or "gut brain axis" or mitochondria* or cost or "cost effective*" or "cost-effective*").tw. 15309040 |
| 6 1 or 2 802712 |
| 7 3 or 4 64917 |
| 8 5 and 6 and 7 2085 |
| 9 limit 8 to yr="2004 -Current" 1656 |
