# Supplementary material for: The Impact of Young and/or Exercised Blood Plasma Transfusions in Individuals With Neurodegenerative Conditions: Protocol for a Scoping Review
Source: JMIR Res Protoc. 2025 Aug 19;14:e65935. doi: 10.2196/65935 (PMC12405790; doi:10.2196/65935)
Supplement: Multimedia Appendix 3 [file resprot_v14i1e65935_app3.docx]

| Study # | Authors, country, and year | Aims, Purpose, Objectives | Sample size | Participants | Condition/s | Age | Type of plasma used and # of treatment sessions | Follow up duration | Key findings related to the scoping review questions |
| --- | --- | --- | --- | --- | --- | --- | --- | --- | --- |
|  |  |  |  |  |  |  |  |  |  |
|  |  |  |  |  |  |  |  |  |  |
|  |  |  |  |  |  |  |  |  |  |
