# Supplementary material for: The Impact of Young and/or Exercised Blood Plasma Transfusions in Individuals With Neurodegenerative Conditions: Protocol for a Scoping Review
Source: JMIR Res Protoc. 2025 Aug 19;14:e65935. doi: 10.2196/65935 (PMC12405790; doi:10.2196/65935)
Supplement: Multimedia Appendix 4 [file resprot_v14i1e65935_app4.docx]

Reports assessed for eligibility:

(n = 2)

Records screened:

(n = 2227)

Reports sought for retrieval:

(n = 235)

Reports assessed for eligibility:

(n = 235)

Studies included in review:

(n = 18)

Records identified from

Databases: (n = 3644)

Registers (n = 13)

Other sources (n = 2)

Records removed before screening:

Duplicate records removed (n = 1432)

Records identified from:

Citation searching (n = 2)

**Identification of studies via databases and registers**

**Identification of studies via other methods**

**Identification**

**Screening**

**Included**

Reports sought for retrieval:

(n = 2)

Records excluded:

(n = 1992)

Reports not retrieved:

(n = 0)

Reports excluded: 219

Wrong study design (n = 69)

Young/exercised plasma not specified (n = 123)

Full text not in English (n= 21)

Wrong patient population (n =2)

Published paper included instead of dissertation (n = 2)

All relevant studies in review already included (n= 2)
